# Anti-CD74 autoantibodies in axial spondyloarthritis as biomarkers for activity and severity of disease but not for tumour necrosis factor inhibitor retention: data from the Swiss Clinical Quality Management in rheumatic diseases cohort

**DOI:** 10.1007/s10067-025-07393-0

**Published:** 2025-03-10

**Authors:** Annik Steimer, Andrea Götschi, Torsten Witte, Almut Scherer, Jonas Brändli, Michael J. Nissen, Burkhard Möller, Simon Grosswiler, Diego Kyburz, Diana Dan, Andrea Rubbert-Roth, Sabine Adler, Oliver Distler, Xenofon Baraliakos, Adrian Ciurea

**Affiliations:** 1 Department of Rheumatology, University Hospital Zurich, University of Zurich, Zurich, Switzerland; 2 Swiss Clinical Quality Management Foundation, Zurich, Switzerland; 3 Department of Rheumatology and Immunology, Hannover Medical School, Hannover, Germany; 4 Department of Rheumatology, Geneva University Hospital, Geneva, Switzerland; 5 Department of Rheumatology and Immunology, Inselspital, Bern, Switzerland; 6 Swiss Ankylosing Spondylitis Association, Zurich, Switzerland; 7 Department of Rheumatology, University Hospital Basel, University of Basel, Basel, Switzerland; 8 Department of Rheumatology, Lausanne University Hospital (CHUV), University of Lausanne, Lausanne, Switzerland; 9 Department of Rheumatology, Cantonal Hospital St. Gallen, St. Gallen, Switzerland; 10 Department of Rheumatology, Cantonal Hospital Aarau, Aarau, Switzerland; 11 Rheumazentrum Ruhrgebiet Herne, Ruhr-University Bochum, Bochum, Germany

**Keywords:** Axial spondyloarthritis, Cohort study, IgA anti-CD74 antibodies, TNF inhibitor treatment

## Abstract

**Objectives:**

Anti-CD74 antibodies (Abs) have been proposed as a diagnostic biomarker in axial spondyloarthritis (axSpA). The aims of this study were to evaluate the association of these Abs with disease activity parameters in axSpA and to assess their predictive value for tumour necrosis factor inhibitor (TNFi) treatment effectiveness.

**Methods:**

Patients diagnosed with axSpA in the Swiss Clinical Quality Management registry with available biosamples and a measurement of IgA anti-CD74 Abs were included in this cohort study. We used a cut-off of 15 U/ml to define anti-CD74 Abs elevation. Associations of important disease characteristics with anti-CD4 Abs elevation and anti-CD74 Abs levels were evaluated using logistic and linear regression, respectively. For patients with an available biosample before TNFi initiation, we evaluated drug retention and estimated the hazard ratio of treatment discontinuation depending on anti-CD74 Abs elevation.

**Results:**

Elevated IgA anti-CD74 Abs were found in 383/722 (53%) patients with axSpA and were significantly associated with older age, male sex, and elevated C-reactive protein (CRP). Among 310 patients starting TNFi treatment, no significant difference in drug retention was found between patients with and without elevated anti-CD74 Abs (HR 0.91, 95% CI 0.66 to 1.25). An increased Bath Ankylosing Spondylitis Disease Activity Index was found to be associated with a reduced TNFi retention whereas an elevated CRP was associated with a prolonged retention.

**Conclusions:**

Although elevated IgA anti-CD74 Abs are associated with CRP elevation, we could not demonstrate an additional value of this biomarker for predicting response to treatment with TNFi beyond CRP measurement.

**Supplementary Information:**

The online version contains supplementary material available at 10.1007/s10067-025-07393-0.

**Table:** 

Key Points • Elevated IgA anti-CD74 antibodies are associated with raised CRP • Elevated IgA anti-CD74 antibodies are not associated with prolonged TNF inhibitor retention after covariate-adjustment		

## Introduction

Autoantibodies against Cluster of Differentiation 74 (anti-CD74 Abs) have emerged as a potential diagnostic biomarker in axial spondyloarthritis (axSpA) [1, 2]. CD74 is the human leucocyte (HLA) class II gamma chain, also known as the invariant chain [2–4]. Anti-CD74 Abs have been shown to cause B-cell proliferation and survival in-vitro [2, 4]. The extracellular domain of CD74 also serves as the cognate receptor of macrophage migration inhibitory factor (MIF), a cytokine potentially involved in the pathogenesis of axSpA through promoting of osteoblastic activity and proinflammatory cytokines [3, 4]. However, the mechanisms leading to the appearance of immunoglobulin A (IgA) and immunoglobulin G (IgG) anti-CD74 Abs in axSpA remain unclear [5, 6]. The sensitivity and specificity of anti-CD74 Abs for axSpA were improved by using the IgA isotype and were found to be of greater value for radiographic than nonradiographic axSpA [7–11]. While anti-CD74 IgA Abs concentration do not appear to correlate with disease activity as assessed by the Bath Ankylosing Spondylitis Disease Activity Index (BASDAI) [10, 12], they are associated with more severe spinal damage and may predict structural progression [9, 10].

Currently, C-reactive protein (CRP) is the only biomarker used in clinical practice to guide the initiation of biologic or targeted-synthetic disease-modifying antirheumatic drugs (b/tsDMARDs) in patients who have an inadequate response to nonsteroidal anti-inflammatory drugs [13]. Indeed, compared to MRI-detected inflammation of the sacroiliac joints, CRP elevation has been shown to better predict treatment response [13].

The aim of this study was to investigate the potential association of elevated IgA anti-CD74 Abs with disease activity parameters, specifically BASDAI and CRP. Additionally, we sought to determine whether these antibodies could provide added predictive value beyond other established predictors in forecasting response to treatment with tumour necrosis factor inhibitors (TNFi).

## Materials and methods

### Study population

Adult patients diagnosed with axSpA by their treating rheumatologist and registered in the Swiss Clinical Quality Management (SCQM) cohort [14] were included in the current study if a serum sample was available in the SCQM biobank (see supplementary material). Clinical assessments conducted during routine care following the recommendations of the Assessment of SpondyloArthritis international Society [15], were recorded in the online SCQM database. The subset of axSpA patients with an available serum sample before the initiation of a TNFi were included in the analysis of treatment retention. The study was approved by the Ethics Commission of the Canton of Zurich (KEK-ZH Nr. 2014–0439) and written informed consent was obtained from all patients before inclusion in SCQM.

### Laboratory assessments

Data on CRP values and HLA-B27 status were collected by the treating rheumatologist and entered in the SCQM online database. IgA antibodies against CD74 were quantified from serum samples available in the SCQM biobank with SpADetect ELISA of Aesku.Diagnostics (Wendelsheim, Germany). Complete recombinant human CD74, produced in HEK293 cells, was used as the antigen. A cut-off value of 15 U/ml was employed, identified as optimal through receiver operating characteristics curve analysis [12].

### Effectiveness of TNF inhibitor treatment

We utilized drug retention as a proxy for assessing TNFi effectiveness. In patients with more than one TNFi treatment course, the first course following the blood sample collection was selected. Patients without a recorded treatment stop date were censored at their last visit date or at the end of the study, whichever came first.

### Statistical analysis

Baseline characteristics at the start of treatment were compared using the Mann–Whitney-U-Test for continuous variables and Fisher’s exact test for categorical variables. No correction for multiple testing was performed.

For the cross-sectional analysis of disease characteristics we used the first measurement of anti-CD74 Abs. The association of disease characteristics with IgA anti-CD74 Ab elevation (dichotomized) or concentration (continuous) was analysed using logistic or linear regression models, respectively (see supplementary material).

We used a Kaplan Meier curve to depict TNFi drug retention, comparing patients with and without elevated IgA anti-CD74 Abs. A log-rank test was conducted to assess the difference in treatment duration. A post hoc-power analysis was conducted based on the estimated hazard ratio with confidence interval for an alpha level of 5%. A multiple adjusted Cox proportional hazards regression model was implemented to estimate a covariate-adjusted association of elevated anti-CD74 Abs on drug retention (see supplementary material).

## Results

Serum samples for the measurement of IgA anti-CD74 Abs were available for 722 patients. Elevated IgA anti-CD74 Abs were detected in 383 (53%) patients. Characteristics of patients with and without elevated anti-CD74 Abs are shown in Table 1. Patients with elevated anti-CD74 Abs were in a higher proportion of male sex (65.3% vs. 51.0%), were older (mean (SD) age 46.4 (12.3) years vs. 41.6 (11.9) years) and had longer symptom duration (median (IQR) 14.3 (7.5; 22.1) vs. 9.9 (5.2; 18.2)). A significantly higher proportion of patients were already treated with a TNFi in the group with elevated anti-CD74 Abs (71.5% vs. 57.2% in the not elevated anti-CD74 Abs group). There were no differences between the groups regarding HLA-B27 status, BASDAI, Axial Spondyloarthritis Disease Activity Score (ASDAS) or functional impairment (assessed by the Bath Ankylosing Spondylitis Functional Index (BASFI) and the Short Form questionnaire with 12 questions (SF-12)). Median CRP (IQR) values were higher (3.9 (1.0; 8.0) vs. 2.4 (1.0; 7.2)) and the impairment of spinal mobility according to the median BASMI (Bath Ankylosing Spondylitis Metrology Index, IQR) more severe in patients with elevated anti-CD74 Abs (1.3 (0.0, 3.0) vs. 1.0 (0.0, 2.0)).

**Table 1:** Characteristics at collection date of all patients with anti-CD74 antibody measurement and baseline characteristics at TNFi start

	Population with available anti-CD74 Abs status				Population with available anti-CD74 Abs status at start of TNFi			
	N	Anti-CD74 Abs elevated	Anti-CD74 Abs not elevated	p-val	N	Anti-CD74 Abs elevated	Anti-CD74 Abs not elevated	p-val
n	722	383	339		310	163	147	
Male sex, N (%)	722	250 (65.3)	173 (51.0)	< 0.001	310	101 (62.0)	67 (45.6)	0.004
HLA-B27 positive, N (%)	652	243 (70.6)	220 (71.4)	0.86	284	101 (66.9)	86 (64.7)	0.71
Age, years	722	46.4 (12.3)	41.6 (11.9)	< 0.001	310	47.8 (11.9)	42.1 (12.2)	< 0.001
Obesity, N (%)	631	69 (20.7)	48 (16.2)	0.15	257	24 (17.4)	16 (13.4)	0.40
Symptom duration, years (median [IQR])	705	14.3 [7.5, 22.1]	9.9 [5.2, 18.2]	< 0.001	304	15.4 [9.2, 22.6]	11.3 [6.2, 18.6]	0.01
Radiographic disease status, N (%)	377	164 (77.4)	110 (66.7)	0.03	161	73 (79.3)	46 (66.7)	0.10
BASDAI (median [IQR])	471	3.7 [1.6, 5.6]	3.8 [1.8, 5.7]	0.81	162	5.1 [3.0, 6.6]	5.1[3.5, 6.5]	0.62
ASDAS	420	2.4 (1.0)	2.3 (1.1)	0.32	125	3.0 (1.0)	2.8 (1.0)	0.57
CRP (mg/l) (median [IQR])	553	3.9 [1.0, 8.0]	2.4 [1.0, 7.2]	0.01	169	5.4 [1.1, 15.0]	3.6 [1.0, 8.9]	0.20
Elevated CRP, N (%)	553	75 (26.6)	59 (21.8)	0.20	169	44 (47.3)	29 (38.2)	0.28
BASFI (median [IQR])	452	1.7 [0.5, 4.5]	1.6 [0.5, 3.7]	0.43	133	4.3 [1.4, 5.9]	2.9 [1.3, 4.9]	0.07
BASMI (median [IQR])	512	1.3 [0.0, 3.0]	1.0 [0.0, 2.0]	0.001	120	2.0 [0.0, 4.0]	1.0 [0.0, 3.0]	0.09
SF-12 PCS (median [IQR])	410	44.4 [32.5, 52.3]	43.3 [33.5, 51.1]	0.74	100	33.6 [28.5, 45.0]	35.6 [30.8, 43.5]	0.54
SF-12 MCS (median [IQR])	410	49.9 [37.9, 55.2]	48.2 [36.1, 55.6]	0.32	100	45.1 [34.0, 52.2]	42.9 [33.9, 51.3]	0.80
EQ-5D (median [IQR])	453	75.3 [59.8, 77.9]	71.3 [62.4, 77.9]	0.28	131	68.7 [46.6, 76.6]	68.7 [48.1, 77.9]	0.39
Peripheral arthritis, N (%)	657	94 (27.6)	86 (27.2)	0.93	245	46 (36.5)	33 (27.7)	0.17
Enthesitis, N (%)	655	175 (51.5)	157 (49.8)	0.70	242	75 (60.5)	70 (59.3)	0.90
Uveitis ever, N (%)	635	98 (29.6)	49 (16.1)	< 0.001	269	44 (31.7)	16 (12.3)	< 0.001
Psoriasis ever, N (%)	556	21 (7.7)	22 (7.7)	1.00	242	13 (11.0)	11 (8.9)	0.67
IBD ever, N (%)	610	31 (9.8)	31 (10.5)	0.79	256	10 (7.6)	14 (11.3)	0.39
Current TNFi, N (%)	722	274 (71.5)	194 (57.2)	< 0.001	310	163 (100)	147 (100)	NA
Previous TNFi, N (%)	722	310 (80.9)	214 (63.1)	< 0.001	310	134 (82.2)	91 (61.9)	< 0.001
Current csDMARD, N (%)	722	64 (16.7)	56 (16.5)	1.00	310	35 (21.5)	17 (11.6)	0.02
Previous csDMARD, N (%)	722	145 (37.9)	99 (29.2)	0.02	310	44 (29.9)	72 (44.2)	0.01
Current NSAID, N (%)	638	227 (68.8)	217 (70.5)	0.67	277	102 (71.8)	100 (74.1)	0.69
Smoker ever, N (%)	640	200 (59.5)	166 (54.6)	0.23	275	86 (60.6)	73 (54.9)	0.39

Presented statistics are means (standard deviation), except where otherwise indicated

*Abs* = antibodies, *ASDAS* = Ankylosing Spondylitis Disease Activity Score, *BASDAI* = Bath Ankylosing Spondylitis Disease Activity Index, *BASFI* = Bath Ankylosing Spondylitis Functional Index, *BASMI* = Bath Ankylosing Spondylitis Metrology Index, *CRP* = C-reactive protein, *csDMARD* = conventional disease-modifying antirheumatic drug, *EQ-5D* = EuroQol 5-domain, *HLA-B27* = Human Leucocyte Antigen B27, *IBD* = inflammatory bowel disease, *NSAID* = nonsteroidal anti-inflammatory drug, *SF-12* = short form 12, PCS = physical component score, *MCS* = mental component score, *TNFi *= Tumour Necrosis Factor inhibitor

### Association of elevated anti-CD74 antibodies with disease activity parameters

In unadjusted logistic regression models, elevated anti-CD74 Abs were associated with older age, male sex, radiographic disease status, uveitis, increased BMI (body mass index), BASMI, and current treatment with bDMARDs. No evidence was found for an association between elevated anti-CD74 Abs and BASDAI, elevated CRP values and HLA-B27 positivity (Table 2). After multiple adjustment elevated anti-CD74 Abs were additionally associated with elevated CRP (OR 2.00, 95% CI 1.11 to 3.68, p = 0.02), but not with higher BASMI and increased BMI, Table 2. The results were confirmed in the adjusted linear regression models, which revealed an association between CRP values and anti-CD74 Abs concentration (Exp (B) 1.11, 95% CI 1.03 to 1.20, p = 0.01), Supplementary Table 1. The exponential regression coefficient (Exp (B)) is the expected multiplicative change in the median of anti-CD74 Abs when increasing the log-transformed CRP by one.

**Table 2:** Association of IgA anti-CD74 antibody elevation with disease characteristics based on logistic regression models

	Unadjusted models			Multiple adjusted model		
Variable	OR	95% CI	p-value	OR	95% CI	p-value
BASDAI	0.99	0.92 to 1.07	0.84	0.96	0.85 to 1.08	0.48
Elevated CRP	1.30	0.88 to 1.93	0.19	**2.00**	**1.11 to 3.68**	**0.02**
BASMI	**1.17**	**1.06 to 1.30**	**0.003**	1.04	0.89 to 1.22	0.58
Male sex (reference: female sex)	**1.80**	**1.34 to 2.44**	** < 0.001**	1.60	0.94 to 2.74	0.08
Age	**1.03**	**1.02 to 1.05**	** < 0.001**	**1.03**	**1.01 to 1.05**	**0.01**
BMI	**1.04**	**1.01 to 1.07**	**0.02**	1.03	0.98 to 1.08	0.30
HLA-B27	0.96	0.69 to 1.35	0.82	0.91	0.52 to 1.59	0.73
Current bDMARD	**1.85**	**1.35 to 2.54**	** < 0.001**	**1.70**	**1.05 to 2.77**	**0.03**
Radiographic disease status	**1.71**	**1.08 to 2.70**	**0.02**			
ASDAS	1.09	0.90 to 1.31	0.38			
BASFI	1.05	0.98 to 1.14	0.20			
Peripheral arthritis	1.02	0.72 to 1.44	0.92			
Uveitis ever	**2.19**	**1.49 to 3.24**	** < 0.001**			
IBD ever	0.92	0.55 to 1.56	0.76			
SF-12 PCS	1.00	0.99 to 1.02	0.74			
SF-12 MCS	1.01	0.99 to 1.03	0.22			
EQ-5D	1.00	0.99 to 1.01	0.70			

Statistically significant results are shown in bold. The unadjusted models were based on the available number of patients per variable as mentioned in Table 1. The adjusted model was based on 319 patients.* ASDAS* = Ankylosing Spondylitis Disease Activity Score, *BASDAI* = Bath Ankylosing Spondylitis Disease Activity Index, *BASFI* = Bath Ankylosing Spondylitis Functional Index, *BASMI* = Bath Ankylosing Spondylitis Metrology Index, *bDMARD* = biological disease-modifying antirheumatic drug, *BMI *= body mass index, *CRP* = C-reactive protein, *EQ-5D* = EuroQol 5-domain, *HLA-B27* = Human Leucocyte Antigen B27, *IBD* = inflammatory bowel disease, *SF-12* = short form 12, *PCS *= physical component score,* MCS* = mental component score

### Drug retention analysis

A total of 310 patients with known anti-CD74 Abs status started treatment with TNFi at any time point after blood collection. A comparable proportion of patients had elevated anti-CD74 Abs in this population (52.6%). Baseline patient characteristics at treatment start stratified by anti-CD74 Abs status are shown in Table 1. Median time (IQR) between anti-CD74 Abs measurement and start of TNFi treatment was 491 days (56.3; 1194.5).

**Fig. 1:**
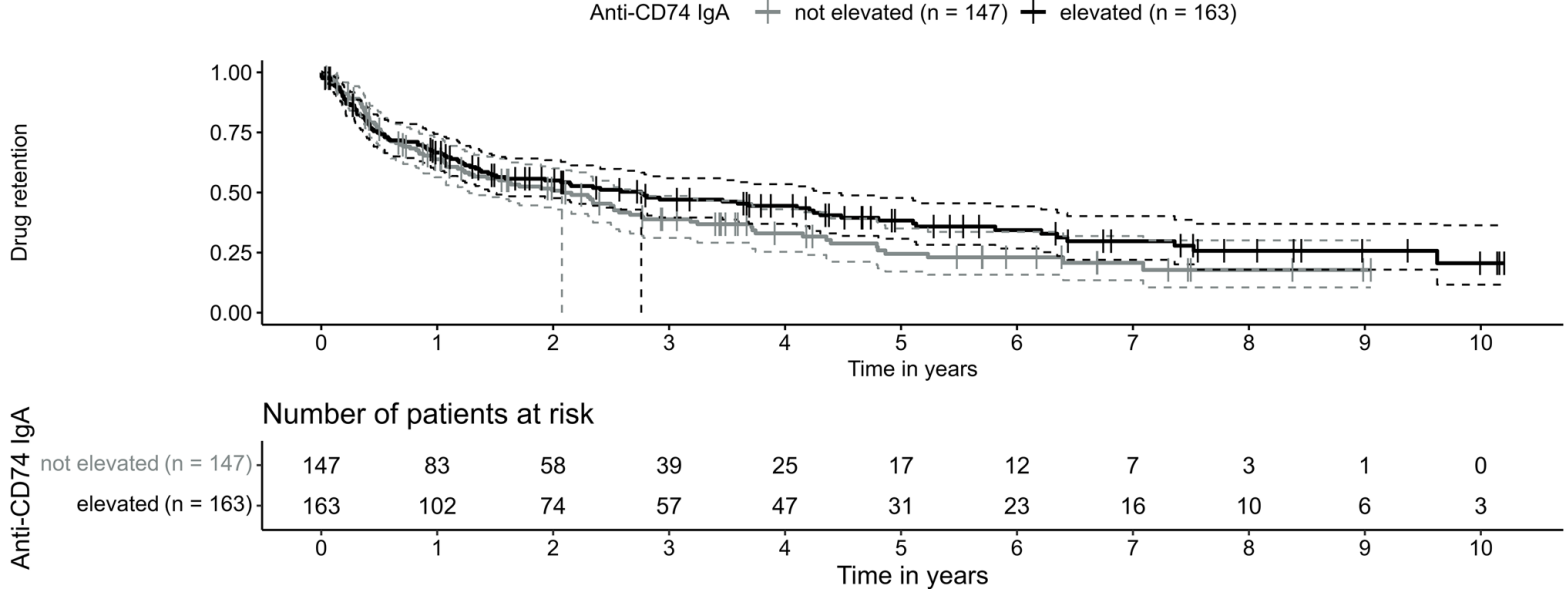
Kaplan–Meier curve of tumour necrosis factor inhibitor retention. Estimated survival curve of patients with elevated IgA anti-CD74 antibodies and patients without elevated IgA anti-CD74 antibodies. Median retention times are represented by vertical lines. 65 patients with elevated and 53 without elevated IgA anti-CD74 antibodies were censored. Anti-CD74 IgA = Anti-CD74 Immunoglobulin A

There was a trend for a longer TNFi retention in patients with elevated anti-CD74 Abs (median (95% CI) 1008 days (537 to 1572) vs 758 days (466 to 1010) in patients without elevated anti-CD74 Abs (log rank p = 0.17; Fig. 1). In the post-hoc power analysis the power for the drug retention analysis was between 9–88%.

For the multivariate analysis, a Cox proportional hazards model adjusted for age, sex, HLA-B27 status, BASDAI, elevated CRP, and previous bDMARD was established. We could not detect a significant difference in drug retention between patients with and without elevated anti-CD74 Abs at start of their TNFi: HR 0.99, 95% CI 0.60 to 1.62, p = 0.96 in the complete case analysis and HR 0.91, 95% CI 0.66 to 1.25, p = 0.55, after imputation of missing variables (Table 3). Moreover, drug retention was not associated with increasing concentration of anti-CD74 Abs at start of TNFi: HR 1.03, 95% CI 0.99 to 1.07, p = 0.13, in the complete case analysis and HR 1.00, 95% CI 0.99 to 1.02, p = 0.75, after multiple imputation of missing covariates (Table 3). BASDAI and CRP were associated with a significantly longer and shorter drug retention, respectively (Table 3).

**Table 3:** Hazard ratios of drug retention estimated using a multiple adjusted Cox proportional hazard model

Variable	HR	95% CI	p-value	HR	95% CI	p-value
	Complete case analysis			After multiple imputation		
**Anti-CD74 Abs elevation**						
Anti-CD74 Abs elevation	0.99	0.60 to 1.62	0.96	0.91	0.66 to 1.25	0.55
BASDAI	1.21	1.08 to 1.36	0.001	1.22	1.11 to 1.34	< 0.001
Elevated CRP	0.47	0.27 to 0.80	0.01	0.60	0.40 to 0.90	0.01
Male sex (reference: female sex)	0.84	0.52 to 1.34	0.46	0.95	0.69 to 1.31	0.74
Age	0.98	0.96 to 1.00	0.08	0.99	0.98 to 1.00	0.15
HLA-B27	0.91	0.54 to 1.53	0.72	1.01	0.71 to 1.45	0.95
Second treatment-line	0.91	0.52 to 1.60	0.75	0.87	0.59 to 1.29	0.50
Third treatment-line	0.87	0.47 to 1.60	0.66	1.02	0.68 to 1.53	0.92
**Anti-CD74 Abs concentration**						
Anti-CD74 Abs (U/ml)	1.03	0.99 to 1.07	0.13	1.00	0.99 to 1.02	0.75
BASDAI	1.22	1.09 to 1.37	0.001	1.23	1.11 to 1.36	< 0.001
Elevated CRP	0.43	0.25 to 0.74	0.002	0.60	0.40 to 0.91	0.02
Male sex (reference: female sex)	0.76	0.47 to 1.23	0.27	0.92	0.67 to 1.27	0.62
Age	0.98	0.96 to 1.00	0.04	0.99	0.98 to 1.00	0.13
HLA-B27	0.87	0.52 to 1.46	0.60	1.01	0.71 to 1.43	0.97
Second treatment-line	0.81	0.46 to 1.43	0.46	0.86	0.58 to 1.26	0.44
Third treatment-line	0.70	0.37 to 1.33	0.28	0.97	0.65 to 1.47	0.90

Complete-case analyses included 121 patients. The models after imputation of missing data were based on 310 patients. In the upper part the model comparing patients with and without elevated antibodies is shown. The results of the model shown in the lower table part included anti-CD74 antibodies as continuous variable. Treatment-line refers to previous bDMARD.* Abs* = antibodies, *BASDAI* = Bath Ankylosing Spondylitis Disease Activity Index, *CRP* = C-reactive protein, *HLA-B27* = Human Leucocyte Antigen B27

## Discussion

Although a decade has passed since the initial detection of anti-CD74 Abs in patients with axSpA, their value as a biomarker in routine clinical practice remains unclear. These autoantibodies appear to lack sufficient diagnostic capacity in early disease states [7, 8], which continue to present significant diagnostic challenges [16]. The lack of prevention of structural spinal progression is another critical area of focus on the research agenda for improving treatment outcomes [17]. Anti-CD74 Abs have been associated with the presence of syndesmophytes [9, 10]. In addition they were shown to predict a faster disease progression in the subsequent two years in an unadjusted analysis [9]. We have now demonstrated an association of antibodies against CD74 with CRP. Given that CRP is a predictor of radiographic progression [18] anti-CD74 Abs seem to be a marker of disease severity. In line with this concept, anti-CD74 Abs have been associated with impairment of spinal mobility in previous studies [9, 19]. While we confirm this association in an unadjusted analysis, it isn't significant after adjustment for potential confounders, indicating that the decrease in spinal mobility is already captured by other factors. We also found an association between the presence of anti-CD74 antibodies and uveitis, an extra-musculoskeletal manifestation of axSpA known to be more prevalent in the radiographic disease state as well as in HLA-B27 positive individuals [20].

Regarding its association with markers of systemic inflammation (CRP) we presumed a possibly better treatment response in patients with elevated anti-CD74 Abs. Therefore we sought to determine whether anti-CD74 Abs might also help in predicting treatment response. Our results suggest that anti-CD74 Abs do not provide additional value beyond CRP and other established predictors, and may, therefore, not be useful as a theragnostic biomarker.

Although patients with elevated anti-CD74 Abs seem to have a higher disease severity, with systemic inflammation (CRP) and extra-musculoskeletal manifestations (uveitis), the quality of health as noted by the EuroQol 5-domain questionnaire was not reduced in patients with elevated anti-CD74 Abs.

The strength of our analysis lies in the relatively large patient population compared to previously published studies on this topic, which all involved fewer than 300 patients [9–12, 21].

However, several limitations of our study must be acknowledged. Although we adjusted our analyses for potential confounders, the possibility of residual confounding cannot be ruled out due to the observational nature of our study. Additionally, as the data was derived from a national European cohort, it remains uncertain whether these results can be generalized to other populations worldwide. Another limitation is the absence of a predefined time frame for blood sampling prior to treatment initiation. Furthermore, missing data on validated outcome assessments prevented an analysis of treatment responses. As a result, we used drug retention analysis as a proxy for treatment effectiveness. Concerning the large range of power for this analysis, it is possible that our study was underpowered. The validation of our results in large multinational cohorts is therefore warranted.

The potential future usefulness of anti-CD74 Abs in clinical practice relates to the fact that CD74 also serves as a receptor for MIF, a cytokine promoting osteoblastic activity [3]. Although TNFi have been shown to retard spinal radiographic progression [22], the inability to prevent structural damage remains one of the major challenges in the research agenda [17]. Since anti-CD74 Abs appear to be associated with disease activity and severity, their potential role in identifying those patients who may benefit from early immunosuppressive treatment to prevent the initiation of osteoproliferative changes requires further investigation. This is particularly relevant in regions with limited or delayed access to magnetic resonance imaging.

In conclusion, although IgA anti-CD74 Abs are associated with markers of systemic inflammation, we could not demonstrate any additional value of this biomarker for predicting treatment response beyond other predictors currently used in routine clinical practice.

## Supplementary Information

Below is the link to the electronic supplementary material.

ESM 1 (DOCX 32.5 KB)

## Data Availability

Restrictions apply to the availability of the data. The data are owned by a third party, the Swiss Clinical Quality Management in Rheumatic Diseases (SCQM) Foundation. Data may be obtained after approval and permission from the license holder (SCQM).
