# Supplementary material for: Anti-CD74 autoantibodies in axial spondyloarthritis as biomarkers for activity and severity of disease but not for tumour necrosis factor inhibitor retention: data from the Swiss Clinical Quality Management in rheumatic diseases cohort
Source: Clin Rheumatol. 2025 Mar 10;44(4):1589–96. doi: 10.1007/s10067-025-07393-0 (PMC11993484; doi:10.1007/s10067-025-07393-0)
Supplement: Supplementary file 1 — (DOCX 32.5 KB) [file 10067_2025_7393_MOESM1_ESM.docx]

Anti-CD74 autoantibodies in axial spondyloarthritis as biomarkers for activity and severity of disease but not for tumour necrosis factor inhibitor retention rate: data from the Swiss Clinical Quality Management in rheumatic diseases cohort

**Supplementary Material**

Annik Steimer^1^, Andrea Götschi^2^, Torsten Witte^3^, Almut Scherer^2^, Jonas Brändli^2^, Michael J. Nissen^4^, Burkhard Möller^5^, Simon Grosswiler^6^, Diego Kyburz^7^, Diana Dan^8^, Andrea Rubbert-Roth^9^, Sabine Adler^10^, Oliver Distler^1^, Xenofon Baraliakos^11^, Adrian Ciurea^1^

^1^Department of Rheumatology, University Hospital Zurich, University of Zurich, Zurich, Switzerland, ^2^Swiss Clinical Quality Management Foundation, Zurich, Switzerland,
^3^Department of Rheumatology and Immunology, Hannover Medical School, Hannover, Germany, ^4^Department of Rheumatology, Geneva University Hospital, Geneva, Switzerland, ^5^Department of Rheumatology and Immunology, Inselspital, Bern, Switzerland, ^6^Swiss Ankylosing Spondylitis Association, Zurich, Switzerland, ^7^Department of Rheumatology, University Hospital Basel, University of Basel, Basel, Switzerland, ^8^Department of Rheumatology, Lausanne University Hospital (CHUV), University of Lausanne, Lausanne, Switzerland, ^9^ Department of Rheumatology, Cantonal Hospital St. Gallen, St. Gallen, Switzerland, ^10^ Department of Rheumatology, Cantonal Hospital Aarau, Aarau, Switzerland,^11^ Rheumazentrum Ruhrgebiet Herne, Ruhr-University Bochum, Bochum, Germany

Corresponding author details:

Annik Steimer,

Department of Rheumatology, University Hospital Zurich,

Rämistrasse 100, CH-8091 Zurich, Switzerland

Email: annik.steimer@usz.ch

ORCiD: 0009-0009-5896-2230

**1. Supplementary information on SCQM register and biobank**

The SCQM axSpA cohort was initiated in 2005 [1]. The central SCQM biobank is located at the Department of Genetic and Laboratory Medicine of the University Hospital of Geneva (HUG) [2, 3]. SCQM provides all materials required for taking blood and for the transportation of the biological specimens at -18°C from the individual rheumatological institutions and private practices to the biobank, where they are further processed, aliquoted, and stored at – 80°C. The samples for this study were drawn between 2011 and 2018. The current study considered clinical data up until 1st February 2024.

**2. Supplementary information on statistical analysis of association with disease activity parameters**

In addition to the unadjusted models, the regression models for disease activity markers (such as BASDAI and CRP) were adjusted for potential confounders, chosen according to previous literature and expert opinion (age, sex, body mass index (BMI), HLA-B27 status, Bath Ankylosing Spondylitis Metrology Index (BASMI), and current treatment with bDMARDs (yes/no). In previous studies an association between anti-CD74 Abs and HLA-B27 status, impaired spinal mobility, and BASDAI has been described [4-7]. Anti-CD74 Abs and CRP were log-transformed using the natural logarithm in the linear regression models. For the association analysis between disease activity and antibodies, all patients with available data on disease activity, regardless of TNFi treatment initiation were included. The closest available BASDAI and CRP were mapped to the blood sample collection date for antibody measurement if the value was assessed within the period starting from the initiation or discontinuation of a new ongoing b/tsDMARD, but no more than 90 days before and 10 days after the blood collection date. Patients that had no measurement of CRP or BASDAI during this time frame were excluded from the disease activity analysis.

**3. Supplementary information on statistical analysis of drug retention analysis**

The following baseline parameters were included in the model as explanatory variables: age, sex, HLA-B27 status, BASDAI, elevated CRP status, and the line of bDMARD treatment (second or ≥3rd versus first-line treatment). An additional model was developed to estimate drug retention rates based on the concentration of anti-CD74 Abs [8] for which we also calculated the adjusted Cox proportional hazard ratios. Missing values were imputed using Multiple Imputation by Chained Equations (MICE) assuming a missing-at-random data pattern [9]. Sixty-one percent of all patients included in the drug retention analysis had at least one missing covariate (HLA-B27 8.4%; BASDAI 47.7%; CRP 45.5%); therefore, 60 imputations and 20 iterations were used in the model. Predictive mean matching was employed to impute continuous variables, while logistic regression was used for binary variables. The following predictors were considered in the imputation model: all explanatory variables included in the Cox proportional hazard model, time-to-event variable (time to TNFi discontinuation or time to censoring) transformed using the cumulative survivor function, a binary variable indicating whether the treatment stop was observed [10], BMI, smoking (not for HLA-B27), current NSAID medication, use of the mySCQM application (only for BASDAI), education level (not for HLA-B27), and radiographic disease status. The treatment line was also included in our imputation model, as clinical visits close to treatment start were more frequently missing in patients initiating treatment in later lines compared to bio-naïve patients. Elevated CRP and BASDAI scores were passively imputed by using the predicted continuous CRP values and individual BASDAI components, respectively. After imputation, we assessed convergence using trance plots and compared the distribution of imputed values to the available data. Model estimates were pooled according to Rubin’s rule [9] and compared to the results obtained in the analyses including only patients with available data (complete-cases). Multiple imputation was performed using the R package MICE [9] in RStudio (R version 4.4.0) [11].

1. Ciurea A, Scherer A, Exer P, Bernhard J, Dudler J, Beyeler B, et al. (2013) Tumor necrosis factor α inhibition in radiographic and nonradiographic axial spondyloarthritis: results from a large observational cohort. Arthritis Rheum 65:3096-3106. <https://doi.org/10.1002/art.38140>.

2. Martinez-Prat L, Nissen MJ, Lamacchia C, Bentow C, Cesana L, Roux-Lombard P, et al. (2018) Comparison of serological biomarkers in rheumatoid arthritis and their combination to improve diagnostic performance. Front Immunol 9:1113. <https://doi.org/10.3389/fimmu.2018.01113>.

3. Scholz GA, Leichtle AB, Scherer A, Arndt U, Fiedler M, Aeberli D, et al. (2019) The links of hepcidin and erythropoietin in the interplay of inflammation and iron deficiency in a large observational study of rheumatoid arthritis. Br J Haematol 186:101-112. <https://doi.org/10.1111/bjh.15895>.

4. Witte T, Köhler M, Georgi J, Schweikhard E, Matthias T, Baerlecken N, et al. (2020) IgA antibodies against CD74 are associated with structural damage in the axial skeleton in patients with axial spondyloarthritis. Clin Exp Rheumatol 38:1127-1131.

5. Abdelaziz MM, Gamal RM, Ismail NM, Lafy RA, Hetta HF (2021) Diagnostic value of anti-CD74 antibodies in early and late axial spondyloarthritis and its relationship to disease activity. Rheumatology (Oxford) 60:263-268. <https://doi.org/10.1093/rheumatology/keaa292>.

6. Hu CJ, Li MT, Li X, Peng LY, Zhang SZ, Leng XM, et al. (2020) CD74 auto-antibodies display little clinical value in Chinese Han population with axial spondyloarthritis. Medicine (Baltimore) 99:e23433. <https://doi.org/10.1097/md.0000000000023433>.

7. Ziade NR, Mallak I, Merheb G, Ghorra P, Baerlecken N, Witte T, et al. (2019) Added value of anti-CD74 autoantibodies in axial spondyloarthritis in a population with low HLA-B27 prevalence. Front Immunol 10:574. <https://doi.org/10.3389/fimmu.2019.00574>.

8. Denz R, Timmesfeld N (2023) Visualizing the (causal) effect of a continuous variable on a time-to-event outcome. Epidemiology 34:652-660. <https://doi.org/10.1097/ede.0000000000001630>.

9. van Buuren S, Groothuis-Oudshooren K (2011) mice: Multivariate Imputation by Chained Equations in R. J Stat Softw 45:1-67. <https://doi.org/10.18637/jss.v045.i03>.

10. Austin PC, White IR, Lee DS, van Buuren S (2021) Missing data in clinical research: a tutorial on multiple imputation. Can J Cardiol 37:1322-1331. <https://doi.org/10.1016/j.cjca.2020.11.010>.

11. R Core Team. R: A language and environment for statistical computing. Vienna, Austria: R foundation for statistical computing; 2024.

**Supplementary Table 1.** Association of IgA anti-CD74 antibody concentration with disease characteristics based on linear regression models

| Variable | Exp (B) | 95% CI | p-value | Exp (B) | 95% CI | p-value |
| --- | --- | --- | --- | --- | --- | --- |
|  | Unadjusted | | | Adjusted | | |
| BASDAI | 0.99 | 0.96 to 1.01 | 0.36 | 0.98 | 0.95 to 1.02 | 0.35 |
| Log (CRP+1) | **1.08** | **1.02 to 1.14** | **0.01** | **1.11** | **1.03 to 1.20** | **0.01** |
| BASMI | **1.04** | **1.01 to 1.08** | **0.01** | 1.02 | 0.98 to 1.07 | 0.35 |
| Male sex  (reference: female sex) | **1.25** | **1.14 to 1.38** | **< 0.001** | **1.20** | **1.02 to 1.41** | **0.02** |
| Age | **1.01** | **1.00 to 1.01** | **< 0.001** | 1.00 | 1.00 to 1.01 | 0.21 |
| BMI | **1.01** | **1.00 to 1.02** | **0.02** | 1.01 | 1.00 to 1.03 | 0.08 |
| HLA-B27 | 1.05 | 0.94 to 1.18 | 0.38 | 1.06 | 0.90 to 1.25 | 0.50 |
| Current bDMARD | **1.27** | **1.14 to 1.40** | **< 0.001** | **1.21** | **1.05 to 1.40** | **0.01** |
| Radiographic disease status | **1.23** | **1.06 to 1.43** | **0.01** |  | | |
| ASDAS | 1.01 | 0.95 to 1.08 | 0.65 |  | | |
| BASFI | 1.01 | 0.98 to 1.03 | 0.61 |  | | |
| Peripheral arthritis | 1.04 | 0.93 to 1.17 | 0.46 |  | | |
| Uveitis ever | **1.32** | **1.17 to 1.50** | **< 0.001** |  | | |
| IBD ever | 0.97 | 0.81 to 1.16 | 0.75 |  | | |
| SF-12 PCS | 1.00 | 1.00 to 1.01 | 0.65 |  | | |
| SF-12 MCS | 1.00 | 1.00 to 1.01 | 0.27 |  | | |
| EQ-5D | 1.00 | 1.00 to 1.00 | 0.47 |  | | |

Statistically significant results are shown in bold. The unadjusted models were based on the available number of patients per variable as mentioned in Table 1. The adjusted model was based on 319 patients. Exp (B) = exponential regression coefficient (Exp B is the expected multiplicative change in the median of anti-CD74 antibodies when increasing the log-transformed CRP by one. For the interpretation of the other covariates in the model, Exp (B) can be interpreted as follows: If a patient has an initial expected median anti-CD74 Abs value of 50 U/ml and the patient would be 5 years older (Exp (B) of age 1.01) the new expected median value of continuous anti-CD74 Abs would increase by the factor of 1.01^5 = 1.05 and therefore would be 52.5 U/ml.). ASDAS = Ankylosing Spondylitis Disease Activity Score, BASDAI = Bath Ankylosing Spondylitis Disease Activity Index, BASFI= Bath Ankylosing Spondylitis Functional Index, BASMI = Bath Ankylosing Spondylitis Metrology Index, bDMARD = biological disease-modifying antirheumatic drug, BMI = body mass index, CRP = C-reactive protein, EQ-5D = EuroQol 5-domain, HLA-B27 = Human Leucocyte Antigen B27, IBD = inflammatory bowel disease, SF-12 = short form 12, PCS = physical component score, MCS = mental component score.
